# Neural representations of visual categories are dynamically tailored to the discrimination required by the task

**DOI:** 10.1093/cercor/bhaf212

**Published:** 2025-08-06

**Authors:** Marlene Poncet, Paraskevi Batziou, Ramakrishna Chakravarthi

**Affiliations:** Department of Psychology, University of Essex, Wivenhoe Park, Colchester, CO4 3SQ, United Kingdom; School of Psychology, University of Aberdeen, King's College, Aberdeen, AB24 3FX, United Kingdom; School of Psychology, University of Aberdeen, King's College, Aberdeen, AB24 3FX, United Kingdom

**Keywords:** discrimination, neural representations, object recognition, task effect, visual categorization

## Abstract

Object categorization is essential to navigate everyday life. It is ultra-rapid, can be completed by purely feedforward mechanisms, and is therefore thought to rely on neural representations that are robust. But how do these representations adapt when category boundaries change (eg buying fruit versus buying apples)? We tested this by asking participants to categorize images at different levels of abstraction while measuring their scalp electrical activity (EEG) with high temporal resolution. Participants categorized images either at the superordinate (animal/non-animal) or at the basic (bird/non-bird) level. We compared classification accuracy and representational similarity of EEG signals between birds, non-bird animals, and vehicles to determine if neural representations are modified according to categorical requirements. We found that neural representations of birds and non-bird animals were indistinguishable in the superordinate task but were separable in the basic task from ~250 ms. On the other hand, the separability of neural representations between non-bird animals and vehicles did not differ by task. These findings suggest that top-down influences modulate categorical representations as needed, but only if discrimination is difficult. We conclude that neural representations of categories are adaptively altered to suit the current task requirements.

## Introduction

Humans quickly recognize objects in their environment. An important aspect of visual recognition is the ability to sort objects into categories based on shared properties such that even newly encountered objects can be recognized (Mervis and Rosch 1981; DiCarlo et al. 2012; Goldstone et al. 2013; Tacchetti et al. 2018; Almeida et al. 2023). Neural representations of object categories need to be stable to enable rapid, consistent, and accurate recognition. However, they also need to be flexible to account for changes in the environment (illumination, appearance, point-of-view, etc.) and support the changing goals of the observer (which category is relevant). In this study, we investigated whether and how categorizing objects at different levels of abstraction might influence neural representations of object categories.

Visual categorization has been extensively studied in humans and non-humans using a range of methods from behavior to single cell recordings and neuroimaging (Epstein and Baker 2019; Bracci and Op De Beeck 2023; Robinson et al. 2023). Such studies have shown that humans can saccade toward a prespecified category in just 120 ms (Kirchner and Thorpe 2006; Crouzet et al. 2010) and categorize complex visual scenes in <300 ms using manual responses (Thorpe et al. 1996; Rousselet et al. 2003; Joubert et al. 2007; Macé et al. 2009; Fabre-Thorpe 2011). fMRI studies have revealed that visual categorization relies on neural processing in the ventral visual pathway (Haxby et al. 2001; Kiani et al. 2007; Kriegeskorte et al. 2008; Grill-Spector and Weiner 2014; Rosenke et al. 2020; Contier et al. 2024) and that regions in the lateral-occipitotemporal cortex show specialized responses to object categories such as faces, tools, places, bodies, and letter strings (Kanwisher et al. 1997; Epstein and Kanwisher 1998; Chao et al. 1999; Cohen et al. 2000; Downing et al. 2001; Haxby et al. 2001). More generally, brain responses to visual objects contain sufficient information to distinguish different object exemplars and, more interestingly, also contain conceptual information about the category of the object. For example, magnetoencephalography (MEG) and electroencephalography (EEG) responses to animal images are distinguishable from those evoked by non-animal images as early as between 80 and 150 ms after stimulus onset (Thorpe et al. 1996; Carlson et al. 2013b; Cichy et al. 2014; Ritchie et al. 2015). Similarly, categorical information can be decoded as early as 100 ms from intracranial field potentials in human visual cortex (Liu et al. 2009) and from single neuron activity in V4/PIT of monkeys (Cauchoix et al. 2016).

The focus of most of the above studies was to determine where (fMRI studies) and when (M/EEG studies) information about object categories is represented in the brain. Surprisingly, however, most of these studies did not require participants to perform a categorization task. Nevertheless, object categories seem to be processed and represented in the brain automatically, regardless of the participants’ task (Groen et al. 2016; see also Delorme et al. 2018), which might explain why real-world objects only require minimal attention to be categorized (Li et al. 2002; Rousselet et al. 2002; Fei-Fei et al. 2005; Poncet et al. 2012). Indeed, Ritchie et al. (2015) found that classification performance for animate and non-animate images based on the MEG signals they evoked was the same irrespective of whether participants performed an animate/non-animate task or an orthogonal letter categorization task (vowel vs. consonant). Similarly, while fMRI studies have found that tasks do affect brain responses to object categories, these effects are mainly observed in the prefrontal cortex with at best relatively small effects of task on representations in the ventral stream (Erez and Duncan 2015; Bracci et al. 2017; Vaziri-Pashkam and Xu 2017; Long et al. 2018; Vaziri-Pashkam and Xu 2019; Xu and Vaziri-Pashkam 2019). Confirming these results, the activity of category selective neurons in the infero-temporal cortex of monkeys do not reflect the category boundary between stimuli unlike in the prefrontal cortex (Freedman et al. 2003; Meyers et al. 2008; McKee et al. 2014). It has thus been suggested that the ventral stream is not involved in decision-related processing, but that its main function would be to create object representations that are stable and robust.

On the other hand, other studies have reported an effect of task on the distributed responses of neurons in the ventral visual stream. For example, Emadi and Esteky (2014) found that category selectivity of macaque IT neurons improved when the monkeys performed a visual shape categorization task compared to a passive fixation condition. Similarly, Sereno and Lehky (2018) showed that the separability of population responses in the ventral stream between shapes was higher when monkeys attended to the shape rather than the location of the stimuli. In humans, Harel et al. (2014) also found evidence for task-dependent modulation of representations in the ventral temporal (and prefrontal) cortex. In their experiment, they asked human participants to categorize the same eight object categories in six different tasks. fMRI multivoxel responses in the early visual cortex allowed comparable classification of object categories within and between tasks, indicating task-independent representations. However, decoding performance was higher when discriminating categories within than between tasks in the ventral temporal and prefrontal cortex, indicating task-dependent representations. More recently, Nastase et al. (2017) demonstrated that for the same stimuli, attention selectively enhanced the discriminability of response patterns along behaviorally relevant dimensions (better discriminability of actions when the task was to attend to animal behavior, or better discriminability of categories when the task was to attend to animal taxonomy). Thus, there is evidence that the goal of the observer (top-down signals) influences the processing and representation of objects in the ventral temporal cortex.

Task effects on categorical representations seem to be strongly related to attention. In fact, the effect of task in visual areas seems to be more evident when selective attention is involved, which is especially the case when multiple categories are presented simultaneously (Peelen et al. 2009; Peelen and Kastner 2011; Çukur et al. 2013; Kaiser et al. 2016; Bugatus et al. 2017; Battistoni et al. 2018; Keller et al. 2022). One clear example of the effect of selective attention comes from the study by Bugatus et al. (2017). In their experiment, they compared three tasks: an oddball task, a working memory task, and a selective attention task in which participants had to attend to one of the two superimposed objects chosen from different categories and indicate if the stimulus was presented upside down. They found that distributed responses in high-level visual cortex were primarily driven by category, while distributed responses in ventrolateral prefrontal cortex were dominated by task demands. Importantly, attention modulated category representations in high-level visual cortex when the task included an element of visual competition, that is in the selective attention task where two stimuli were superimposed, but not in the other two tasks where only one stimulus was presented at a time. Similarly, using artificial stimuli varying in shape and color, Barnes et al. (2022) found better category decoding (between different shapes or different colors) of the relevant than the irrelevant feature when selective attention was involved, that is, when two stimuli were presented simultaneously compared to if only one stimulus was presented at a time. Thus, attention could selectively exaggerate the distance between neural representations of visual objects along relevant dimensions when multiple objects are presented simultaneously. Some have argued that attention itself can be reframed as task demands (Rosenholtz 2024). Thus, the above findings can be taken to suggest that task demands modify neural representations of categories.

In this study, we directly tested whether and how the discriminability between object categories changes depending on the task while keeping the same object always relevant (ie not manipulating selective attention). For this, we used two very similar categorization tasks that differed only by their level of abstraction (superordinate or basic) instead of changing the nature of the tasks (semantic vs. perceptual) or using tasks that rely on different stimulus attributes, as was done in previous studies (Harel et al. 2014; Nastase et al. 2017). This means that in our study, both tasks rely on neural processing in the same brain areas (category selective areas in higher level visual areas), while in previous studies different tasks could rely on neural processing in different visual areas (eg categorizing the orientation or color of an object).

Superordinate categorization, such as categorizing animals against non-animal objects, is faster and more accurate than basic-level categorization, such as categorizing birds against other animals (Macé et al. 2009; Praß et al. 2013; Poncet and Fabre-Thorpe 2014; Wu et al. 2015; Vanmarcke et al. 2016). This superordinate categorization advantage has been explained as due to categories at the superordinate level (bird vs. car) being more dissimilar compared to those at the basic level where categories are more similar to each other (bird vs. dog) (Bowers and Jones 2008; Kadar and Ben-Shahar 2012; Mohan and Arun 2012). Indeed, using a computational model based on task-specific perceptual discriminability between images, Sofer et al. (2015) could accurately predict behavioral responses in different categorization tasks. Thus, behavioral performance in categorization tasks at different levels of abstraction are tightly related to the discriminability of the stimulus categories.

Interestingly, behavioral performance in categorizing animate from inanimate images is also related to the discriminability of neural representations. MEG studies have found that reaction times in an animate/non-animate categorization task could be predicted by the distance between the neural representation of an object and its task-defined category boundary (the boundary dividing representations of animate and inanimate objects): the closer the neural representation to the task boundary, the longer the RT in the categorization task (Carlson et al. 2013a; Ritchie et al. 2015; see also Bankson et al. 2018). Although such studies highlight the relationship between discriminability of neural representations with categorization speed, they only investigated categories at the superordinate level (animate, inanimate). Some studies (Carlson et al. 2013b; Cichy et al. 2014; Contini et al. 2017) compared neural responses to objects grouped at different level of categorization, but they did not require participants to actively categorize the stimuli (and thus also did not directly correlate RT performance with similarity in neural patterns). Therefore, these results are purely based on passive viewing that might not be directly relevant to behavioral findings. In fact, these studies did not find support for the superordinate advantage seen in behavioral performance. There was very little variability in peak decoding times between individual exemplars and different levels of category abstraction. When considering the representational geometry of the neural representations, Carlson et al. (2013b) found a maximal differentiation of individual exemplars at 120 ms and a distinction between animate and non-animate categories later, at 240 ms. It is possible that the superordinate advantage found in behavioral performance is due to differences in decisional processes and not in how/when categories are represented in the ventral visual pathway. On the other hand, it is also possible that the differences in peak decoding times between different levels of abstraction do not reflect the discriminability of the categories per se, since participants were doing an orthogonal task, but instead might reflect arbitrary stimulus level differences (Badwal et al. 2024).

Differences in the neural representations of object categories might be observable and more relevant when participants are actively performing categorization tasks, particularly those with differing requirements (Nau et al. 2024). As reviewed earlier, the discriminability of neural representations is modulated by attention and task demands. In addition, the abstraction level at which the category of an object is processed seems to be dependent on the current categorization goal of the participant (Poncet et al. 2020). Even when a very briefly presented (20 ms) image should be ignored, its category interferes with the categorization of a subsequent image, but this interference depends on the task: in superordinate animal/non-animal categorization task, a dog prime facilitates the categorization of a target bird image as an animal while in a basic bird/non-bird categorization task, the same dog prime hinders the categorization of the target bird image as a bird. Importantly, in the basic categorization task, a dog prime hinders the categorization of a bird target image more than a vehicle prime does. That is, the distance of the prime object category to the task-defined category boundary affects behavioral performance. This suggests that the neural representation of object categories might be modulated depending on the current discrimination demands.

In this study, we investigated how the level of categorization (superordinate and basic) influences the discriminability of neural representation between three different stimulus categories: birds, non-bird animals, and vehicles. The two categorization tasks require recognizing and categorizing objects and therefore rely on the same visual areas. Our design thus specifically targets how neural responses in the visual pathway to object categories change depending on task demand. At the superordinate level, birds and non-bird animals are both targets and do not need to be discriminated. At the basic level, however, the task requires birds to be discriminated from the other animals. By comparing responses to the three different categories in the two categorization tasks, we can examine when and how the discriminability requirement for a given task might affect behavioral performance and the separability of neural representations.

## Method

The experimental program, original behavioral data, preprocessed EEG data, programs for running the behavioral and EEG data analysis, and their results are available at https://osf.io/q84t3/.

### Participants

Twenty participants (6 males and 14 females, all University undergraduates) took part in the experiment. They had self-reported normal or corrected-to-normal vision and provided written informed consent. The experiment received the approval of the Psychology Ethics Committee, University of Aberdeen (PEC/3893/2018/5).

### Material and stimuli

Participants were seated in a dimly lit room, ~50 cm in front of a CRT monitor (800 × 600 pixel resolution, 41 cm screen width, refresh rate 100 Hz). The stimuli, colored natural images of animals and vehicles, were presented in the center of the screen on a gray background. They were displayed using MATLAB with PsychToolbox extensions (Brainard 1997; Kleiner et al. 2007) with a size of 16° × 16° visual angle and were synchronized with the refresh rate of the monitor. We used a set of 3,072 images used in a previous study (Poncet et al. 2020). Half of the images were birds, a quarter were vehicles, and the final quarter were non-bird animals (mammals and fish). The image presented in a trial was randomly selected from each category and never repeated across the experiment. This allowed us to make sure that any differences that arise in EEG analyses between object categories reflect categorical differences and are not based on spurious differences between individual images. This is particularly problematic when the same images are presented multiple times in the train and test set in EEG decoding analysis. In addition, not repeating images prevent potential effects of stimulus–response associations that could arise (and which might be appropriate or inappropriate for the current task) (Denkinger and Koutstaal 2009; Eckstein and Henson 2012). It has also been shown that humans can hold a large number of images in long-term memory (estimates exceed 2,500 over a session) even when viewed for a short duration (Brady et al. 2008; Konkle et al. 2010; Brady et al. 2011). Thus, it is likely that participants would be able to notice that they are viewing the same images, and potential associations could come into play.

### Procedure

**Fig. 1:**
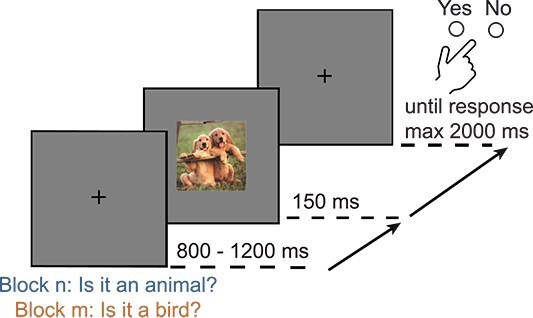
Paradigm. In each trial, participants were asked to categorize an image presented for 150 ms at the superordinate level (animal/non-animal) or at the basic level (bird/non-bird) in separate blocks.

At the beginning of a trial, a black fixation cross was presented at the center of a gray screen for a random interval between 800 and 1,200 ms (Fig. 1). The stimulus then appeared for 150 ms, followed by the fixation cross. At the beginning of each block, participants were informed about the task in that block: they were asked to categorize the stimulus at the superordinate level (whether the image contained an animal) or at the basic level (whether the image contained a bird) by pressing the left (“yes” response) or right (“no” response) arrow key using the same hand. Participants were given feedback on their accuracy and encouraged to aim for high accuracy. A beep was produced if they made a mistake or if they did not answer within 2,000 ms. The next trial started immediately after the participant’s response or after a maximum of 2,000 ms after the onset of the stimulus. Participants performed 10 blocks of 200 trials each, 5 for superordinate categorization and 5 for basic categorization. In superordinate blocks, 50% of the images contained an animal (half of which were birds and the other half non-bird animals) and the remaining 50% were vehicles. In basic blocks, 50% of the images were birds, 25% were other animals, and the remaining 25% were vehicles. The order of the blocks and trials was randomized for each participant. Before the main experiment, participants were trained on 32 trials of each categorization task.

### E‌EG recording and preprocessing

Participants’ EEG was recorded using a 64-channel BioSemi Active Two system at a sampling rate of 1,024 Hz. Four additional external electrodes were used to monitor eye movements and blinks. One was placed below the left eye, one above, and two were placed at the outer canthi of both eyes.

The EEG recordings were preprocessed using EEGLAB (Delorme and Makeig 2004) extensions in MATLAB. The signal was filtered between 0.5 and 48 Hz (using the default parameters for the function eegfiltnew; parameters are listed in Supplementary Materials, section 1.1) and re-referenced to the average. The resulting raw data were cleaned using the inbuilt EEGLAB function clean_rawdata with conservative criteria (see Supplementary Materials, section 1.1). Independent component analysis was applied to the cleaned signal. For each participant, eye-blinks were identified and removed automatically using a 90% confidence threshold. Epochs were created ranging from 150 ms before to 500 ms after stimulus onset. Baseline correction was applied to each channel by subtracting the average activity between −150 and 0 ms relative to stimulus onset. Epochs with a difference of >150 μV between the two vertically placed eye channel electrodes were removed. Noisy channels were interpolated using spherical interpolation. At the end of this process, three participants with very noisy signal (with >40% of trials rejected or >20% noisy channels) were excluded from further analysis. On average, 4 electrodes were interpolated and 273 (13.7%) trials per participants were rejected for the 17 participants included in the final analysis.

All 64 electrodes were used in the EEG analyses. We included both correct and incorrect trials in our analyses since participants’ accuracy was very high in all conditions (the number of trials would be roughly the same for analyses based on all trials vs. those based on correct-only trials). We further reasoned that it is unclear whether an incorrect trial reflects a difference during visual processing stages or during the decision process. Similarly, there could be a certain amount of lapse rate (pressing the wrong button unintentionally). We thus used a conservative approach and included all trials in all EEG analyses. However, to ascertain whether our results were affected by the inclusion of incorrect trials, we ran additional analyses (specifically, MVPA) with only correct trials. The pattern of results was highly similar to that reported here (see Supplementary Materials, section 2.3).

### Data analysis

#### Behavior

We analyzed participants’ accuracy using d-prime (following Hautus 1995 correction) and reaction times (RTs) for correct responses separately for the superordinate and basic-level categorization tasks. Anticipatory responses (RT faster than 200 ms) and trials without any response (no response within 2 s) were excluded from the analysis. To investigate the effect of task on the three categories of interest (bird, non-bird animal, vehicle), we computed RT and hit rates for these three categories separately at each categorization level. We used JASP to perform pairwise *t*-tests between them. All results are reported after Bonferroni correction (total of 3 comparisons).

We ran a Hierarchical Drift Diffusion Model (HDDM; Wiecki et al. 2013) on the behavioral data to identify whether task affects one or more of processes involved in decision making. Specifically, we assessed the effect of task on evidence accumulation (or drift-rate, *v*), decision boundary (*a*), and non-decisional (*t*) processes. The three parameters of the model (*a*, *t*, *v*) were fitted using an accuracy-coding procedure for each of the three object categories with the upper threshold corresponding to correct responses (hit) and the lower threshold to incorrect responses (miss). We evaluated the convergence of the model by visually inspecting the traces of the posteriors, the autocorrelation, the marginal posterior, and by using the Gelman–Rubin diagnostic (Gelman and Rubin 1992) which resulted in *R* values that were all <1.0019. We also performed posterior predictive checks (see Supplementary Materials, section 1.2).

Hypothesis testing was performed by taking advantage of the Bayesian estimation of the DDM parameters provided by the HDDM toolbox. We analyzed the probability, *P*, that two conditions are different from each other by determining the amount of overlap between the posterior distributions of the two conditions (if *P* = 0.5, the two distributions fully overlap; if *P* = 1 or *P* = 0, they do not overlap). In contrast to *P*-values used in frequentist statistics, this analysis provides a direct probability measure, but it can still be interpreted in a similar way as *P*-values.

#### Event-related potential

We wanted to compare our results to an earlier study that used a similar design and reported differences between animal and vehicle images (VanRullen and Thorpe 2001). For this, we averaged the trials for each category within each categorization task to obtain the corresponding event-related potential (ERP), for each participant. We then computed the differential responses between pairs of categories (bird vs. non-bird animal, non-bird animal vs. vehicle, bird vs. vehicle) separately for the two categorization tasks (superordinate and basic). This resulted in six differential waveforms per participant.

To allow comparison with previous results, we averaged the ERPs across electrodes in three ROIs: occipital electrodes (O1 PO3 PO7 Oz O2 PO4 PO8), central electrodes (C3 C1 Cz C2 C4), and frontal electrodes (AF3 F1 AFz Fz F2 AF4), within shortened epochs lasting −50 to 250 ms. The statistical significance of the differential responses between pairs of categories was assessed using cluster-based permutation tests (Maris and Oostenveld 2007) with the “permutest” function (Gerber 2024). Alternative statistical analyses can be found in Supplementary Material (section 2.1.1), as well as ERP results using unfiltered data (section 2.1.2). Overall, outcomes of these additional analyses align with the findings of the study.

#### Decoding

We could evaluate neural representations of categories using multivariate classification procedures, as no two images were the same across the entire experiment. For each participant, we performed multivariate pattern analysis on a trial-by-trial basis for each timepoint of the EEG data. We used linear discriminant classifiers through inbuilt MATLAB functions (fitcdiscr) with 10-fold cross-validation to discriminate between pairs of image categories (three pairs: bird vs. animal, animal vs. vehicle, bird vs. vehicle) for the two categorization tasks separately. The same number of trials per category was randomly sampled and used to train the six classifiers. We repeated this 10 times to sample different sets of trials being included in the analysis (and different subsets for cross-validation). We performed the same analysis but while randomly shuffling the labels of the pairs of conditions to obtain the corresponding baseline (chance) performance. Significance testing was performed by comparing decoding performance and baseline performance (i.e. real vs. shuffled conditions) using cluster-based permutation tests. Using shuffled conditions as a baseline is a more conservative approach than using a fixed 50% chance level (e.g. a fixed baseline has no variance and is easier to be “different” from than a shuffled baseline). Additional cluster-based permutation tests between the decoding performance in the superordinate and the basic tasks for each category pair were performed to examine the effect of task. Alternative statistical analyses were conducted examining the robustness of the findings (Supplementary Materials, section 2.1.1) as well as sensitivity analyses testing the outcomes for a range of sample sizes (section 2.1.3). The latter confirmed that our results are already reliable with a sample of 10 participants. Note that we did not directly compare the same category across the two tasks, as the trials were in separate blocks. Noise levels and patterns might differ across blocks, and these might drive decoding performance (Hebart and Baker 2018; Li et al. 2021) and need not reflect modulation of neural representations.

#### Representational similarity analysis

We followed the procedure outlined by Guggenmos et al. (2018). For each participant, we selected an equal number of trials for each condition (equal to the number of trials in the condition with the fewest trials). For each condition, these trials were then randomly partitioned into five subsets and averaged within each subset to form five pseudotrials. Multivariate noise normalization was applied to these trials at each time point in the epoch. Cross-validated Euclidean distances were then computed between relevant pairs of conditions, at each time point, by training on four pairs of pseudotrials and testing on the remaining pair. This process was repeated 20 times for each participant and the obtained distances were averaged across all iterations to produce a measure of dissimilarity between them. Significance testing was performed using cluster-based permutation tests comparing the cross-validated Euclidean distances against 0. The effect of task was tested by performing cluster-based permutation tests between the cross-validated Euclidean distances in the superordinate and the basic tasks for each category pair.

#### Multidimensional scaling

At selected time points over the epoch (50 to 400 ms in 50-ms steps, and 480 ms), cross-validated Euclidean distances computed above were averaged over a 30-ms window (15 ms on either side of the selected time point) and then represented as dissimilarity matrices for each of the two task levels (superordinate and basic). This dissimilarity matrix was subjected to multidimensional scaling (MDS) using the MATLAB function cmdscale to obtain coordinates in a multidimensional representational space. Coordinates in the first two dimensions were used to plot the relative locations of categories for visualization.

#### Temporal generalization

In addition to the decoding procedure outlined above, we also investigated the generalization of the decoding performance over all timepoints. We used the same method as for the decoding analysis except for the following. To decrease the amount of computation, the EEG signal of each trial was averaged every ~ 4 ms (thus reducing the total number of timepoints by 4). The classifier was then trained at each timepoint (ie −150 to 500 ms in steps of ~ 4 ms) and tested on all timepoints. Significance testing was performed by comparing decoding performance and baseline performance (ie real vs. shuffled conditions) using (2D) cluster-based permutation tests; small nonsignificant timepoints enclosed within a significant cluster were also considered significant using the MATLAB function imfill.

#### Functional source localization

To determine the electrodes involved in discriminating pairs of categories, for each participant and each of the six conditions we transformed the LDA classifier’s weights obtained in the decoding analysis back to an activation pattern following Grootswagers et al. (2017) (see also Haufe et al. 2014). These activation patterns represent the scalp topographies of weights used by the classifier that best discriminate between pairs of categories in the two tasks. Because we used an LDA classifier, these activation patterns are equivalent to the EEG difference between two conditions in mass-univariate analysis (ie they are the same as the difference in ERPs at each electrode between the two conditions that are entered in the decoding analysis) (Haufe et al. 2014).

We further investigated the brain areas involved in discriminating pairs of categories by using a functional source localization method based on EEG templates (Poncet and Ales 2023). The time courses of the classifier’s weights for each the six pairwise decoding conditions (bird vs. non-bird animal, non-bird animal vs. vehicle, bird vs. vehicle, at the superordinate and basic levels) were simultaneously submitted to this analysis. To determine the statistical significance of differences between pairs of categories, we computed null distributions for each pair (1,000 bootstrap samples for each of the 6 comparisons) by applying the template method to classifier’s weights while randomly shuffling the labels of (paired) conditions by randomly assigning a positive or negative sign to the classifier’s weight. We then fitted a Gaussian distribution to the bootstrapped null distributions and determined the *z*-score of the actual data relative to this distribution (for each paired category, each brain area, and time point). We then obtained a *P*-value from the *z*-score using equation 1:


(1) \begin{equation*} P=2\ast \left(1-\mathrm{normcdf}\left(\mathrm{abs}(z)\right)\right) \end{equation*}

where *z* is the *z*-score and normcdf is the cumulative distribution function of the normal distribution. We take any difference with *P* <0.005 (Benjamin et al. 2018) for at least 15 consecutive milliseconds as statistically significant.

## Results

### Behavior

**Fig. 2:**
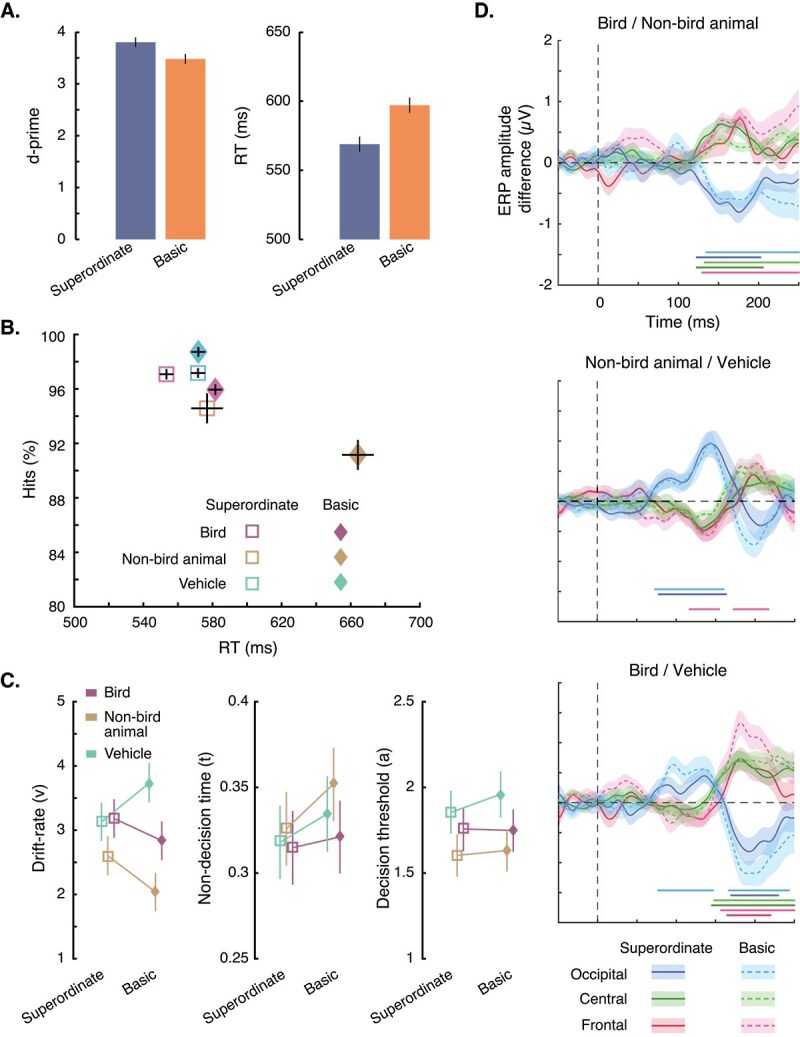
Results of the experiment. Behavioral performance for categorizing the images is plotted by task (A) and by image category (B). Error bars represent 95% within-subject confidence interval (Cousineau 2005; Morey 2008). (C) Estimated HDDM parameters for the different categories of images in the two categorization tasks. Error bars represent 95% credible interval. (D) Difference in ERPs between the three pairs of object categories. Each line plots the difference in responses between two categories for a given categorization task averaged over electrodes located at occipital, central, or frontal sites. Shaded areas around the mean amplitude represent SEM. Note that the time window is shorter than that used in other analyses for easier comparison with previous studies. Significant differences between pairs of categories are represented with matching-colored dots at the bottom of the figures.

Participants were accurate in categorizing natural scene images (Fig. 2A). They were better at the superordinate-level task than at the basic-level both in terms of d-primes (*t*(19) = 5.05; *P* < 0.001; Cohen’s *d* = 1.13) and reaction times (*t*(19) = 7.61; *P* < 0.001; Cohen’s *d* = 1.70), replicating previous findings (Macé et al. 2009; Praß et al. 2013; Poncet and Fabre-Thorpe 2014; Vanmarcke et al. 2016).

Since we are particularly interested in the processing of the three object categories (bird, non-bird animal, vehicle), we also investigated the change in behavioral performance for these three categories across the two categorization levels (Fig. 2B). The results show that performance for categorizing bird images was better at the superordinate than at the basic level for both hits (*t*(19) = 3.27; *P* = 0.012; Cohen’s *d* = 0.73) and RTs (*t*(19) = 8.02; *P* < 0.001; Cohen’s *d* = 1.79). Similarly, categorizing non-bird animal images was also easier (*t*(19) = 3.56; *P* = 0.006; Cohen’s *d* = 1.29) and considerably faster (*t*(19) = 10.89; *P* < 0.001; Cohen’s *d* = 2.43) in the superordinate than in the basic task. Finally, vehicle images were categorized with a higher accuracy in the basic task than in the superordinate task (*t*(19) = 4.91; *P* < 0.001; Cohen’s *d* = 1.10), while categorization speed remained the same (*t*(19) = 0.05; *P* = 1; Cohen’s *d* = 0.01). Better performance at the basic than at the superordinate level might seem surprising and even contradictory to previous results, but the primary bottleneck of the basic-level task is to differentiate between bird and non-bird animal images (as observed in the effect of task on bird and non-bird animal images). Vehicles are on the other hand easier to exclude from the target category.

#### Drift diffusion modeling

The behavioral data discussed above indicate that categorization level affects visual processing of objects. To determine the processing stage at which these differences arise, we modeled the data using HDDM (Fig. 2C). The results suggest that the Criterion parameter, “*a*,” is not affected by the level of categorization (*P*s > 0.13 for the three object categories). Non-decisional processes, *t*, are also not affected by the change between superordinate and basic levels for bird and vehicle categories (*P*s > 0.14). However, they are slower in the basic than in the superordinate categorization task for non-bird animals (*P* = 0.046). This probably reflects the fact that non-bird animals change status from target to distractor across the two tasks, while the other two categories always remain targets (bird) or distractors (vehicle) across tasks. Importantly, for all object categories, the drift-rate parameter “*v*” changes significantly from superordinate to basic level. It becomes slower at the basic level for animal (*P* = 0.0046) and bird (*P* = 0.057) categories while it becomes faster for the vehicle category (*P* = 0.003). These results directly mirror the difficulty in discriminability between the three categories: while birds and non-bird animals become harder to discriminate at the basic level, vehicles, on the other hand, are farther away from the categorization boundary, and hence easier to categorize at the basic level. These results indicate that the rate of evidence accumulation required to categorize images, which likely takes place in higher visual areas, is modulated by the level at which they are categorized.

### Event-related potential

We analyzed the differences in ERPs between the three pairs of categories (birds and non-bird animals, non-bird animals and vehicles, birds and vehicles) in the two categorization tasks. First, we computed these differences in sets of electrodes located in the occipital, central, and frontal regions (Fig. 2D). The ERP difference between bird and non-bird animal images shows significant clusters from ~125 ms after stimulus onset in all three electrode sets and for both tasks. This aligns well with previous studies showing categorical EEG differences around a similar timepoint after stimulus onset (Thorpe et al. 1996; Fabre-Thorpe et al. 2001; VanRullen and Thorpe 2001). Although the differences in ERP responses to non-bird animal and vehicle images are not evident in all electrodes, the overall shape of these ERP differences are impressively similar to that reported in figure 3 of VanRullen et al.’s study (VanRullen and Thorpe 2001). These findings remarkably replicate the >20-year-old result with different images, different EEG setups, and different preprocessing pipelines. Significant clusters starting at ~75 to 80 ms and lasting until 160 ms are visible in occipital electrodes in both superordinate and basic categorization tasks. In their study, VanRullen et al. argued that these early effects were perceptual and task independent (while effects at 125 to 160 ms would reflect categorical differences). This might be a result of, for example, differences in the spatial frequency envelope of animal (natural) and vehicle (man-made) images (Oliva and Torralba 2001). In fact, this early effect is also visible in the ERP difference between bird and vehicle images at the basic level (the effect at the superordinate level is weaker). In addition, when comparing bird and vehicle images, we observe significant clusters from ~145 to 170 ms across all electrodes and tasks. In general, the ERP differences that we report here confirm previous results showing early effects, ~75 to 80 ms, which might be attributable to differences in low-level image statistics between natural and man-made images, while later effects, ~125 to 160 ms, are likely reflecting categorical differences. The ERP results for each electrode separately and for the entire time window from 150 ms before to 500 ms after stimulus onset are reported in Supplementary Material (section 2.2).

### Multivariate analysis

The goal of the current study was to investigate if and how the level of categorization affects the neural representation space of (ie discrimination between) object categories. We expected to find the strongest effect of task between bird and non-bird animal categories: at the superordinate level, bird and non-bird animal images do not need to be discriminated while at the basic level they do.

**Fig. 3:**
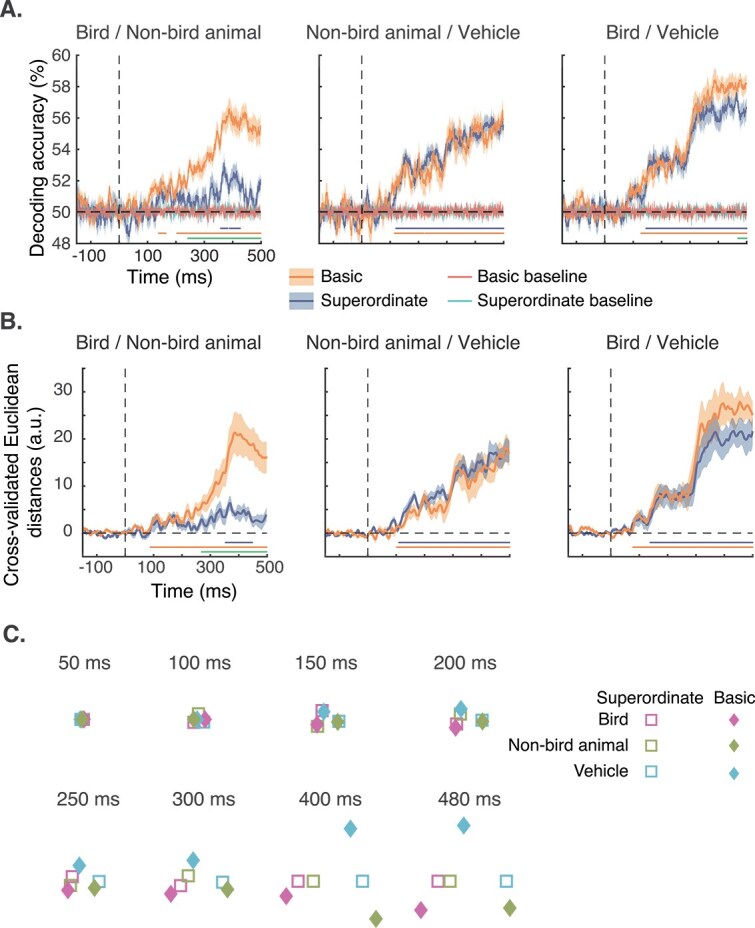
Results of the multivariate analyses. Results of the decoding (A) and the representational similarity (B) analyses between the three pairs of categories either at the superordinate (blue) or at the basic (orange) level. Significant differences compared to baseline decoding performance are represented with matching-colored dots at the bottom of the figures. The green dots represent significant differences between the two categorization tasks. Shaded areas around the mean represent SEM. (C) Representational distances between bird, non-bird animal, and vehicle categories in the two categorization tasks using MDS analysis at different timepoints after stimulus onset. Note that the absolute locations are not relevant as they are 2D projections of the locations of the three categories from a multidimensional space. The relative locations (to each other) are more informative.

Overall, linear discriminant classifiers were able to use the information contained in the EEG signals to allow differentiation between members of all six tested pairs of categories (Fig. 3A). Decoding performance confirmed that neural representations are more similar between bird and non-bird animal at the superordinate level than at the basic-level categorization. In fact, at the superordinate level, the two categories were barely discriminated by the classifier, perhaps only at ~360 ms after stimulus onset, while they could already be classified above the baseline from 140 ms onwards in the basic categorization task (latencies of the cluster-based permutation tests). Cluster-based permutation tests between the two tasks indicated that they were significantly different from 245 ms.

On the other hand, the discriminability of non-bird animal and vehicle neural representations was not affected by the task. These two categories could be decoded better than the baseline from ~120 ms. Furthermore, in both the superordinate and basic categorization tasks, decoding performance was the same. Decoding performance reached an initial plateau at 120 ms and a second higher plateau at ~300 ms. Very similar results were found for the discriminability of bird and vehicle neural representations with slightly noisier decoding performance at early timepoints (with significant cluster differences starting ~130 to 150 ms) and reaching higher decoding performance at the second plateau.

### Representational similarity analysis and multidimensional scaling

Decoding performance is based on a binary prediction of categories and hence a dichotomous measure of whether an image in a given trial was correctly discriminated or not. To obtain further insight into the discriminability of the pairs of categories, we computed the distance between the neural representations of the categories. The pattern of results is similar to that of decoding performance (Fig. 3B). The discriminability of (cross-validated Euclidean distance, or dissimilarity between) bird and non-bird animal representations is higher in the basic categorization task while being close to 0 in the superordinate categorization task. While the neural representations of these two categories are reliably separable with a significant cluster from ~90 ms after stimulus onset in the basic-level task, a cluster is observed only ~355 ms in the superordinate-level task. A significant cluster reflecting the differences between the two tasks can be seen from 275 ms onwards suggesting that the discrimination of bird and non-bird animal images is still relatively noisy before this time.

The distance between neural representations of animal and vehicle categories and that of bird and vehicle categories does not seem to be affected by the level of the categorization task. As with results from our classification analysis, we find two plateaus of discriminability, one between 150 and 300 ms and one between 300 and 500 ms. Distances between bird and vehicle categories seem to be less noisy at the earlier timepoints in the superordinate task and to be higher than that of non-bird animal and vehicle representational distances.

We used MDS on the cross-validated Euclidean distances obtained above to map out the representational space of all three categories in the two tasks (see Fig. 3C for different timepoints after stimulus onset). Descriptively, at 50 ms, and to some extent 100 ms, after stimulus onset, the three categories, within each task, are overlapping and not differentiable. At 150 ms, the three categories are separated but there are no noticeable differences across tasks. Note that the overlap of categories across tasks is not informative; only the relative distance between categories within each task is meaningful. With time, the effect of task becomes evident. The representations of the three categories in the basic task move away from each other from 250 ms, unlike in the superordinate task. The distances among the former keep increasing after 300 ms while they change minimally for the categories in the superordinate task context. In fact, one dimension in the representational space is lost from 400 ms, suggesting that activity along a single dimension is sufficient to separate the three categories. Interestingly, in the superordinate task, bird and non-bird animal category representations are closer to each other than either is with the vehicle category. On the other hand, bird, non-bird, and vehicle appear equidistant from each other in the basic task.

### Temporal generalization

**Fig. 4:**
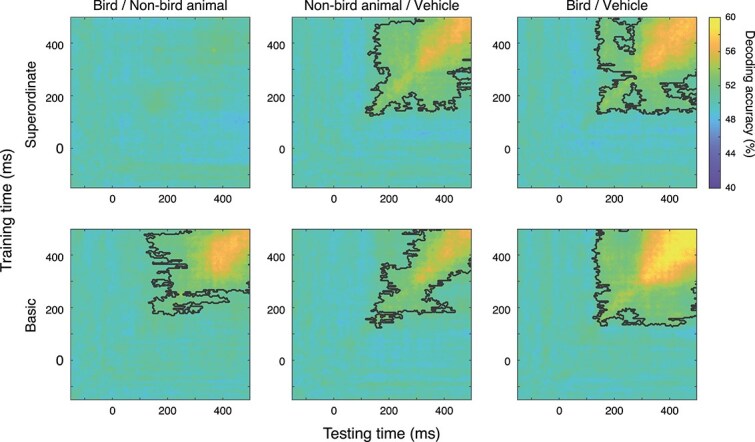
Temporal generalization of decoding accuracy. Decoding performance between the three pairs of categories at the superordinate (top row) and at the basic (bottom row) level when trained and tested at different time points. Points within the black contours are significantly different from baseline decoding performance, as determined by cluster-based permutation tests.

It is possible that the neural information that discriminates categories is transient, and that the extent of this transience might be task dependent. That is, information that allows the classifiers to distinguish categories might change from timepoint to timepoint, and this variability might be higher in one task relative to the other, implying that representations are more stable during one task than the other. To examine this, we performed a temporal generalization analysis of decoding (Fig. 4).

As expected, bird and non-bird animal representations are not separable for the superordinate task, and there is no generalizability. The same categories are nevertheless separable at the basic level from 120 ms onwards. Decoding performance is relatively low until 300 ms but generalizable across most of the epoch, indicating that the information present is stable across time.

On the other hand, temporal generalization of discrimination between non-bird animals and vehicles seems to vary between superordinate and basic levels. The information used to decode the two categories at early timepoints (~120 ms) is comparable to that at later timepoints for superordinate-level categorization but is more transient and does not seem to be similar across timepoints for basic-level categorization; that is, the distinguishing information is more time specific. The early visual category–specific information (from ~120 ms) might not be useful for the basic categorization as non-bird animal and vehicle categories are both distractors (they need to be merged). On the other hand, the same category-specific information might still be useful for superordinate categorization in which non-bird animals are targets and vehicles are distractors (the two categories need to be separated). The difference in the temporal generalization for non-bird animal versus vehicle at the two levels of categorization could thus be explained by a difference in the utility of early categorical information for the task. Note that information in both tasks can be sufficient to provide similar decoding performance along the diagonal (timepoint-specific decoding). That is, information is stable at the superordinate level and transient at the basic level but discriminates equally well at both levels.

The discrimination between bird and vehicle categories does not seem to differ much with the level of categorization, except that decoding accuracy is higher overall at the basic than at the superordinate level (as observed in the other results). The information used to separate the two categories in early stages (~120 ms) is also used at later stages, as in the case of non-bird animal versus vehicle representations for superordinate categorization. This provides converging evidence that early visual information is category specific and task independent and generalize to later timepoints if it is useful for the task (ie if the task requires discriminating between the two categories).

### Functional source localization

**Fig. 5:**
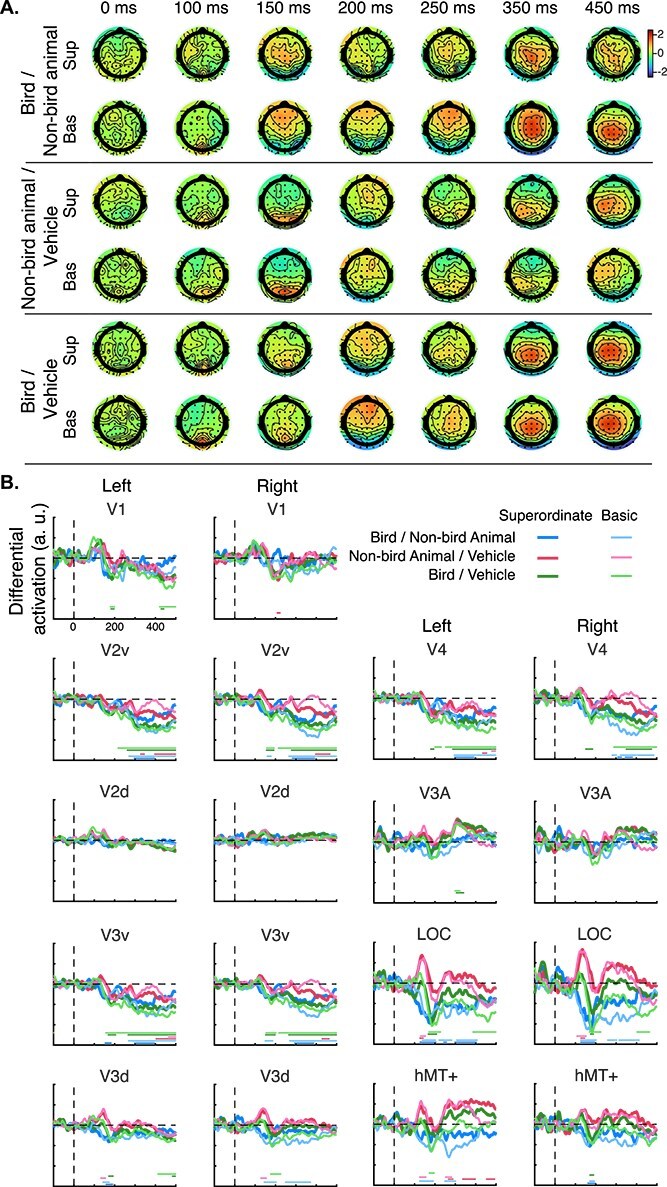
Source localization results. (A) Topographies of each electrode’s contribution to the decoding performance (classifier weights) for each pair of categories in the superordinate (sup) and basic (bas) categorization tasks. (B) Time courses of the contribution of visual areas to discriminating pairs of object categories (one line for each of the six conditions). Significant contributions (*P* < 0.005 for at least 15 ms) are represented with matching-colored dots at the bottom of the figures.

In order to determine the contribution of each of the 64 electrodes to decoding performance, we projected the classifier’s weights back to a scalp activation pattern. The topographies, while relatively similar across conditions (see Fig. 5A), also display differences. To further uncover the visual areas responsible for the observed topographies, we fed the time courses of decoding weights to a functional source localization method based on EEG templates (Fig. 5B). Remarkably similar results are obtained by using ERP differential waveforms for source localization, given their similarity in topographies to that of the classifier weights (Supplementary Materials, section 2.4).

The functional source localization of the classifier’s weights confirmed that processing differences between object categories occurs mainly in extrastriate and higher level visual areas. Overall, the results suggest two stages. First, category differences are processed from ~120 to 150 ms mainly in the LOC (and to a lesser extent by V3v and V3d) from ~150 to 170 ms. This result is consistent with previous studies finding that LOC represents object categories (Kourtzi and Kanwisher 2001; Grill-Spector and Weiner 2014; Bracci and Op De Beeck 2023). In a second stage, from ~270 to 280 ms after stimulus onset, categorical differences are mainly observed in V2v, V3v, and in V4 from 300 ms. Although this effect is clear regardless of the task (superordinate or basic), it is not evident for the difference between non-bird animal and vehicle categories. This second stage might reflect feedback processes to ventral extrastriate areas.

When comparing bird and non-bird animal categories, the topographies at 150 ms are largely similar between the two tasks but the activity is more sustained over time at the basic level (up to 250 ms) than at the superordinate level. Electrode contributions (weight topographies) for the two tasks are noticeably different between 350 and 450 ms, with activations in the basic task similar to that observed between bird and vehicle categories between 350 and 450 ms. These differences seem to be driven by continuous activation (starting from 250 to 500 ms) of V2v, V3v, V4, and LOC in the basic task but not in the superordinate task. These differences in activity could reflect additional feedback processes required in the basic task where the two similar categories need to be separated, unlike in the superordinate task.

Classifier weight topographies are similar between the superordinate and basic tasks for decoding between non-bird animal and vehicle categories. There seems to be some differences in late timepoints (450 ms) driven by differences in V2v and V3v activity between the two categories for the superordinate task that are absent in the basic task.

For discriminating bird and vehicle categories, the topographies are largely the same, except that the weights are higher in the basic than the superordinate task. The waveforms obtained from the functional source localization are highly similar between the two tasks with V2v, V3v, V4, and LOC contributing substantially to the categorical discrimination. The effect of task is mainly observed in LOC from 250 ms.

## Discussion

In this study, we examined whether and how the level at which an object is categorized affects the neural representations of object categories. Behavioral results show that superordinate-level categorization is faster and more accurate than basic-level categorization. This replicates previous findings (Macé et al. 2009; Praß et al. 2013; Poncet and Fabre-Thorpe 2014; Wu et al. 2015; Vanmarcke et al. 2016) and confirms that behavioral performance in categorization tasks reflects the discriminability of object categories: similar categories are more difficult to discriminate than dissimilar ones (Carlson et al. 2013a; Ritchie et al. 2015; Sofer et al. 2015; Ritchie and Carlson 2016; Poncet et al. 2020). Interestingly, the decrease in performance at the basic level was driven by an increase in the difficulty to categorize non-bird animal images and, to some extent, bird images. Drift diffusion modeling of these results suggests that this was primarily due to a slow-down in evidence accumulation toward the non-bird animal category at the basic level compared to the superordinate level. Importantly, several EEG analyses using different approaches all confirmed that the neural representations of these two categories (birds and non-bird animals) change with task demands. When categorizing at the superordinate level, the neural representations of bird and non-bird animal images are very similar. However, when the task is to categorize at the basic level, they are more separated. This task effect is reliable from ~250 ms after the onset of the stimulus, primarily driven by activity in V2v, V3v, V4, and LOC. In contrast, the level of categorization had almost no effect on the separability of neural representations of bird and vehicle categories and between non-bird animals and vehicles. Thus, the results of this study clearly show that task goals affect behavioral and neural responses in a directed manner during rapid visual processing of object categories.

Neural (ERP) responses for bird, non-bird animal, and vehicle categories are very similar but yet do show reliable differences that can be observed starting in the LOC ~130 ms. These effects are likely to reflect the processing of categories in higher level visual areas (Liu et al. 2009; Bracci and Op De Beeck 2023). While pairs of categories could be discriminated from one another, decoding performance was overall very low between bird and non-bird animals in the superordinate task. This is consistent with the drift diffusion modeling results showing a slower drift-rate for processing birds and especially non-bird animals in the basic than in the superordinate task. These results further confirm that the superordinate advantage seen in behavioral responses (in this and previous studies; eg Bowers and Jones 2008; Macé et al. 2009; Sofer et al. 2015) can be explained by the difficulty in separating neural representations in the ventral stream of objects that belong to the same superordinate category during basic-level categorization.

The MDS results show that while between 150 and 200 ms the neural representations of birds, non-bird animals, and vehicles are separated, the effect of the level of categorization task only starts from 250 ms. This effect of task is evident for discriminating bird and non-bird animals. Our results suggest that the fine-grained discrimination required at the basic level to discriminate birds from non-bird animals might be subserved by feedback to early visual areas in the ventral stream (V2v, V3v, V4), likely from higher areas involved in category representation (LOC), from 250 ms. These timings are consistent with those of Hebart et al. (2018) who reported that task-related information could be decoded from MEG signals from ~240 ms while object category–related information could be decoded earlier, ~105 ms. This suggests an initial feedforward processing stage of visual categorization that is largely task independent, which might explain the previously documented similar peak decoding times across different levels of category abstraction (Carlson et al. 2013b; Cichy et al. 2014; Contini et al. 2017), particularly under passive viewing conditions that these studies tested. The temporal generalization results also show that the early information used to discriminate pairs of categories is used at later time points during visual processing. The results further suggest that this generalization is probably the consequence of the utility of this information for the task (for discriminating the two categories). When the two categories do not need to be discriminated but instead need to be merged, as is the case between non-bird animals and vehicles at the basic level, early information is not as clearly generalizable.

An effect of task ~250 ms is also consistent with studies showing an effect of attention on neural representations ~250 ms (Groen et al. 2016; Battistoni et al. 2018; Moerel et al. 2022). For example, Groen et al. (2016) found that while categorical ERP differences between natural and man-made scenes were present from 80 to 200 ms, these differences also recurred after 250 ms when participants actively categorized the images but, interestingly, not when they performed a task on an unrelated superimposed stimulus. Similarly, Battistoni et al. (2018) found that although the presence of the target category could be decoded from 180 ms, the effect of spatial attention, that is, higher decoding of target location than distractor location, emerged only from 240 ms. Thus, categorical differences ~250 ms seem to be related to selective attention enhancing the representation of the attended stimulus category. In our study, there is only one stimulus presented at a time and hence there is no requirement for attention to bias any competition between two stimuli. However, other top-down mechanisms such as in our case task requirements and the necessity to separate bird representations from other animals might influence how object categories are processed and represented in the ventral visual pathway (Kay et al. 2023; Rosenholtz 2024).

We also observed a general increase in the separability of neural representations between pairs of categories from 300 ms, reaching maximum dissimilarity in the representational space (MDS) between representations at 400 ms. These effects, again, possibly originating in early visual areas (V2v, V3v, and V4) might be the consequence of feedback until reaching a decision (RTs are on average ~550 to 600 ms). This suggests that although category-related information might be processed in a feedforward manner, feedback and modification of representations continue to occur at later timepoints. In summary, categorical differences, irrespective of task, are processed by higher visual areas by ~150 to 200 ms. Later, current goals and requirements modify these representations through top-down, possibly feedback, directed mechanisms to extrastriate visual cortex from 250 ms onwards.

The effect of categorization level was predominantly visible on the (task mandated) separability of bird and non-bird animal neural representations which increased during the basic, compared to superordinate, categorization task. The separability of other pairs of categories was not affected by the task. The lack of difference in the separability of representations across categorization levels between animal and vehicle categories indicates that the observed difference among birds and non-bird animals is not merely due to a change in the status of non-bird animals from target (superordinate task) to distractor (basic task). If it were so, we should have expected a similar effect on representations of non-bird animals and vehicles, as non-bird animals change status across tasks relative to vehicles. The same argument applies to differences in motor responses. Indeed, if neural responses were driven by differences in motor responses between target and distractors, we should have found a difference between non-bird animals and vehicles at the superordinate level but not at the basic level. We also note that the lack of discriminability between representations of vehicles and other categories is not due to a floor effect. In addition, if motor responses were driving differences in neural responses, we should observe a systematic latency difference between the superordinate and the basic tasks. Given the strong effect of categorization level on RT for non-bird animal images (~100 ms), this latency effect should be evident in the neural discriminability between non-bird animal representations and vehicle representations as well as in the temporal generalization results as an offset, which we did not observe. Therefore, the changes in discrimination between non-bird animal and bird neural representations cannot be attributed to differences in target status, response preparation, or motor responses.

We did observe an effect of the status (target vs. distractor) of categorical representations in the temporal generalization results. Information distinguishing non-bird animal and vehicle categories was more generalizable at the superordinate level than at the basic level. Furthermore, the distance between the categorical representations, as determined by time-specific decoding and representational similarity analysis (RSA) results, were comparable at both levels of categorization. These results suggest that neural representations, while similarly separable across tasks, are more stable at the superordinate than at the basic level. This might be attributable to the status of the image as target versus distractor: while the two categories are mapped on different responses at the superordinate level, they must be mapped onto the same response at the basic level. Note that this, nevertheless, cannot explain the effect of task on bird and non-bird animal discrimination, where separability is substantially different across the two tasks. A second point to note is that the status of the image is most certainly represented in the brain and most probably in frontal regions (for decision making) as shown in several previous studies (Harel et al. 2014; eg Bracci et al. 2017). However, the method we used (EEG and our analytical approaches) might not be the most suitable for finding effects in frontal regions; indeed, our focus was to investigate modifications of neural representations in visual areas.

We can think of two possibilities that could explain why the effect of task is only observed when comparing bird and non-bird animal categories. One is that the task always induces changes in neural representations but is sometimes not visible because those changes are too small. The second possibility is that the task induces changes in neural representation space only for objects belonging to the same superordinate category (objects that are more similar), not for objects that are very distinguishable. In our case, we could imagine a representational space in which only the bird representations change at the basic level to become separated from the rest of the animals while the distance between other animals and vehicles remains the same. This would explain the absence of an effect of task between other animals and vehicles. The distance between bird and vehicle neural representations might change as well but not necessarily.

Consistent with this idea, in a previous study (Poncet et al. 2020) we found that the interference effect of a vehicle prime on the categorization of a subsequent bird image was relatively small compared to that of a non-bird animal prime, suggesting that the competition in neural representation was relatively weak in the presence of vehicle primes. These findings suggest that the neural representation of vehicles is likely very distinct from the neural representation of animal categories such that the task we used here would not meaningfully change the distance between vehicle and animal neural representations. In addition, it appears that during category-learning, discriminability of neural representations improves mainly for representations of boundary-adjacent exemplars (Ester et al. 2020; O’Bryan et al. 2024). This suggests that neural representations for distant objects do not change even if the task does. Thus, the lack of an effect of categorization level on the discrimination between vehicle neural representations and other categories could illustrate the robustness of category processing in high visual areas. Additional experiments could examine the conditions (ie at which degree of similarity) under which task begins to affect the neural representational space of categories in the visual ventral stream. In general, our results suggest that neural representations of object categories are very stable and are only modified when a task boundary has to be placed between similar representations to classify them.

The higher separability of bird neural representations from other animals at the basic level might be the consequence of higher signal-to-noise ratio of the neural responses during basic categorization. When learning to categorize objects according to new rules, the neural discriminability of object representations in the visual cortex is increased between relevant category dimensions (Folstein et al. 2013). Other category-learning studies using inverted encoding models found that neural representations in early visual cortex become more selective and biased away from the newly learned decision boundary, especially for challenging exemplars near a category boundary (Ester et al. 2020; O’Bryan et al. 2024). It is thus possible that during basic-level categorization, bird representations underwent a similar enhancement or a stronger bias away from other animal representations, leading to better neural discriminability (decoding performance, RSA, MDS) relative to non-bird animal representations. Indeed, Emadi and Esteky (2014) found that during active viewing, compared to passive viewing, neural responses in the IT cortex of monkeys showed a larger firing rate enhancement for the preferred category and smaller response variability for both preferred and nonpreferred categories. Further experiments would be necessary to determine the exact mechanisms underlying the modulation observed in our study.

## Conclusion

In this study, we show that behavioral and neural responses to object categories are affected by task context. Our results demonstrate both robustness in how categories are represented and flexibility in their representational space, depending on current goals. Information about the category of an object is processed rapidly, around the same time, and in the same brain areas regardless of the task. Furthermore, the neural representation of object categories that are easily discriminable do not seem affected by task requirement. On the other hand, neural representations of similar categories that need to be discriminated will be more segregated from each other than when they do not need to be discriminated, likely through feedback to extrastriate areas. This flexibility in representational space allows appropriate behavior corresponding to current goals.

## Supplementary Material

suppCategEEG_final_bhaf212
