# Supplementary material for: Neural representations of visual categories are dynamically tailored to the discrimination required by the task
Source: Cereb Cortex. 2025 Aug 6;35(8):bhaf212. doi: 10.1093/cercor/bhaf212 (PMC12341912; doi:10.1093/cercor/bhaf212)
Supplement: suppCategEEG_final_bhaf212 [file suppcategeeg_final_bhaf212.docx]

Supplementary material

Neural representations of visual categories are dynamically tailored to the discrimination required by the task

Marlene Poncet^1*^, Paraskevi Batziou^2^ and Ramakrishna Chakravarthi^2^

^1^ Department of Psychology, University of Essex, Colchester, UK

^2^ School of Psychology, University of Aberdeen, Aberdeen, UK

Corresponding author: Marlene Poncet

Current address:

Department of Psychology

University of Essex

Wivenhoe Park

Colchester

United Kingdom

Contact: marlene.poncet@essex.ac.uk

# Supplementary methods: Data analysis

## EEG pre-processing

The EEG signal was filtered between 0.5 to 48 Hz using pop_eegfiltnew function. The function automatically determines the filter length based on the Firfilt plugin. It then filters the data using Hamming windowed sinc FIR filter.

For the ‘*pop_clean_rawdata’* function, channels were removed if a) they were flat for more than 5 s b) had a high-frequency noise standard deviation above 6 c) a correlation with nearby channels that was less than 0.8. Data periods with more than 40 standard deviation burst correction for 0.5 s were removed, as well as periods for which the channel power was outside -Inf to 7 range.

## HDDM

We generated 20,000 posterior samples and discarded the first 2,000 samples as burn-in. In addition to inspecting the output of the model and using the Gelman–Rubin diagnostic, we also evaluated the convergence of the model by performing posterior predictive checks, which consist of simulating new data from the fitted model and comparing it to the original data. The observed and predicted RT distributions for correct and incorrect responses in the different conditions are shown in Supp Fig 1 and the summary statistics of the posterior predictive checks can be found in Supp Table 1. They confirm that the predicted RT distribution falls within the 95% credible interval of the observed RT distribution. That is, the model produces data that closely mimics the observed data, indicating that the model fit was excellent.


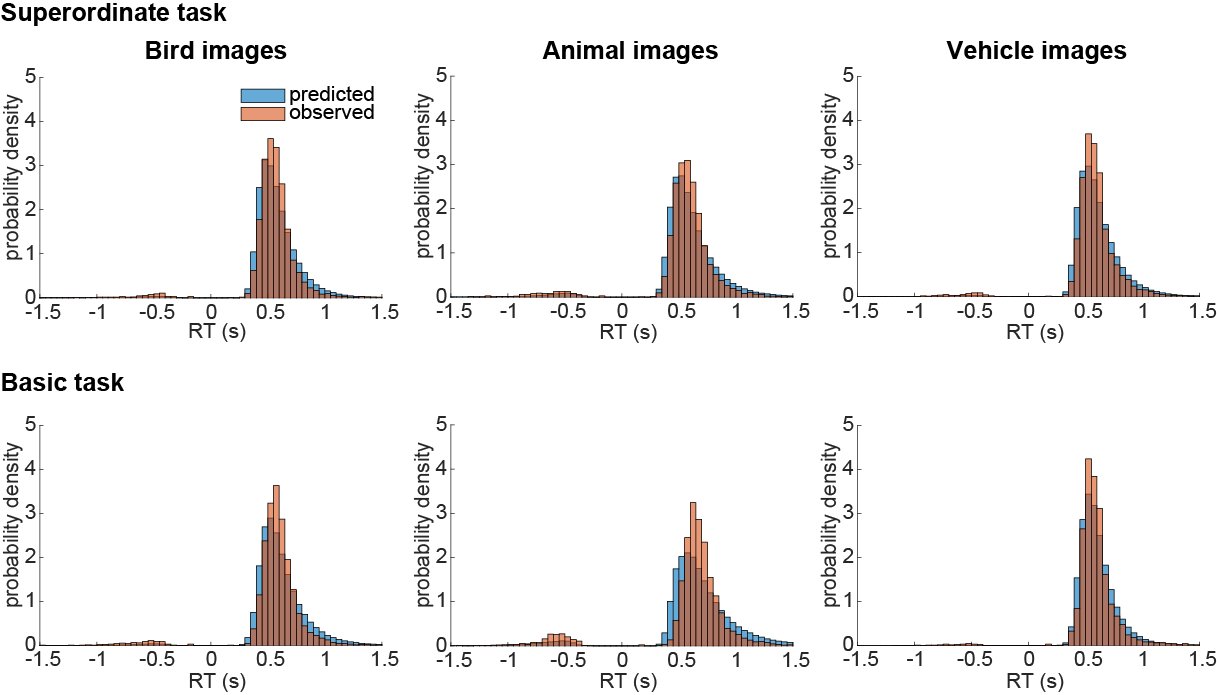


Supplementary Figure 1. Histograms of posterior predictive checks. Observed (red histograms) and HDDM predicted (blue histograms) RT distributions for the different image categories in the superordinate and basic categorisation tasks. Incorrect responses are represented with negative RTs.

|  | Observed | Simulated | STD | SEM | MSE | within 95% credible interval | Mahalanobis distance |
| --- | --- | --- | --- | --- | --- | --- | --- |
| mean accuracy | 0.96 | 0.98 | 0.03 | <.001 | 0.001 | TRUE | 0.75 |
| mean RT upper boundary | 0.61 | 0.63 | 0.07 | <.001 | 0.005 | TRUE | 0.28 |
| std upper boundary | 0.15 | 0.19 | 0.06 | 0.001 | 0.005 | TRUE | 0.66 |
| 10 quartile upper boundary | 0.46 | 0.45 | 0.05 | <.001 | 0.003 | TRUE | 0.20 |
| 30 quartile upper boundary | 0.53 | 0.51 | 0.05 | <.001 | 0.003 | TRUE | 0.27 |
| 50 quartile upper boundary | 0.58 | 0.58 | 0.06 | <.001 | 0.004 | TRUE | 0.03 |
| 70 quartile upper boundary | 0.65 | 0.68 | 0.08 | 0.001 | 0.007 | TRUE | 0.37 |
| 90 quartile upper boundary | 0.79 | 0.87 | 0.13 | 0.006 | 0.024 | TRUE | 0.61 |
| mean RT lower boundary | -0.62 | -0.64 | 0.13 | <.001 | 0.017 | TRUE | 0.12 |
| std lower boundary | 0.21 | 0.15 | 0.11 | 0.004 | 0.017 | TRUE | 0.56 |
| 10 quartile lower boundary | 0.41 | 0.51 | 0.12 | 0.008 | 0.022 | TRUE | 0.78 |
| 30 quartile lower boundary | 0.50 | 0.55 | 0.11 | 0.003 | 0.016 | TRUE | 0.49 |
| 50 quartile lower boundary | 0.58 | 0.60 | 0.12 | 0.001 | 0.016 | TRUE | 0.24 |
| 70 quartile lower boundary | 0.69 | 0.68 | 0.15 | <.001 | 0.023 | TRUE | 0.05 |
| 90 quartile lower boundary | 0.90 | 0.81 | 0.23 | 0.008 | 0.062 | TRUE | 0.39 |

Supplementary Table 1. Summary statistics of the posterior predictive checks. The first and second columns represent the observed and simulated mean values for each measure indicated by the row name. The upper boundary represents correct responses while the lower boundary represents incorrect responses. STD is a measure of how much variation is produced in the summary statistics. The rest of the columns are various measures of the extent of deviation between the simulated and the observed data (SEM is the standard error from the mean, MSE is the mean-squared error).

# Supplementary results

## Sensitivity analyses for the EEG results

### Alternative statistical test

In this additional analysis, we tested the statistical significance of the differential responses between pairs of categories using paired t-tests. To correct for multiple comparisons while being able to accurately report the latency of a potential effect (Sassenhagen and Draschkow 2019; but see Rousselet 2025), we consider any difference with p<.005 (Benjamin et al. 2018) for at least 15 ms as statistically significant.

This is a relatively conservative measure: there only needs one data point with p> .005 within 15 ms for the significant p-values to not be reported. A good example of this is when comparing decoding performance between non-bird animal and vehicle images in Fig. S3. Although decoding performances are similar for the two tasks, they fluctuate more in the basic task such that significant p-values (compared to baseline) are only reported at 250, and then at 300 ms while they are reported at around 150 ms in the superordinate task.

Overall, the statistical results are similar to the ones found with cluster-based permutation tests. Categorical differences in ERPs are visible around 125-160 ms in most electrodes and for all tasks (Fig. S2). These differences are weaker when comparing non-bird animal and vehicle categories and later when comparing bird to vehicle categories. Latencies of the decoding performance are later when comparing bird and non-bird animal category representations, and in the basic task between non-bird animal and vehicle categories (Fig. S2). RSA results are similar to that in the main analyses with only some additional significant differences between bird and non-bird animal categories at earlier timepoints (Fig. S3).


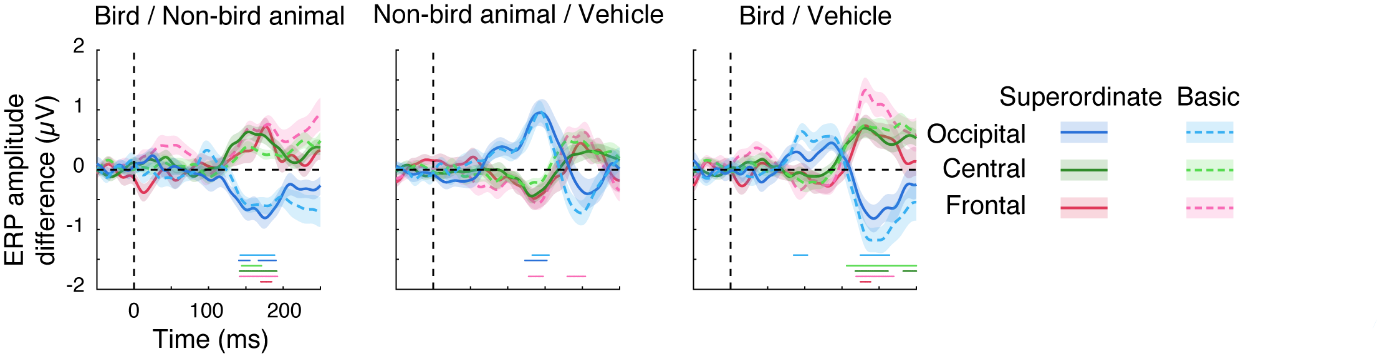


Supplementary Figure 2. Alternative statistical results using significant differences for p<.005 for at least 15 ms as a criterion. Difference in ERP responses between the three pairs of object categories are plotted as in Fig. 2 (main text). Significant differences are represented with coloured dots at the bottom of the figures.


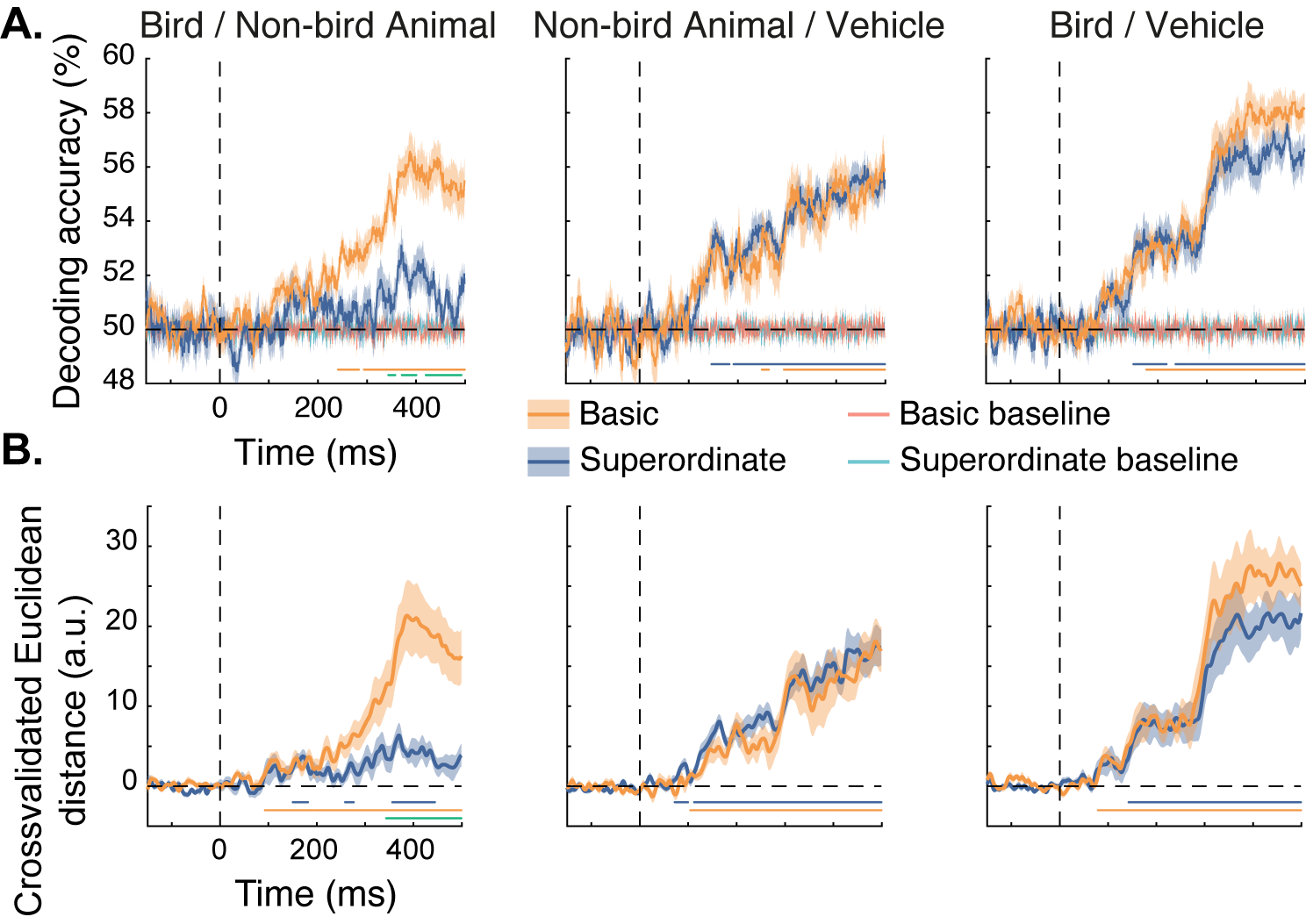


Supplementary Figure 3. Alternative statistical results using significant differences for p<.005 for at least 15 ms as a criterion. A. Decoding performance and B. results of the representational similarity as in Fig. 3 (main text). In all panels, significant differences are represented with matching coloured dots at the bottom of the figures. Green dots indicate differences across tasks.

### Unfiltered ERP results

To confirm that the latency differences in the ERP analyses were not affected by filtering the EEG data (VanRullen 2011; Tanner et al. 2015) we additionally analysed the results using non-filtered EEG data. As in the main analysis, we computed the ERP responses for each category over three set of electrodes (occipital, central, frontal) and computed the difference between the three pairs of object categories for the two tasks separately. To test the significance of the differential responses we used both cluster-based permutation tests (as in the main analyses) and paired t-tests with p<.005 for at least 15 ms.

The ERP waveforms are highly similar to that in the main analyses (Fig. S4). There are some differences in latencies depending on the statistical test, but they are overall consistent.


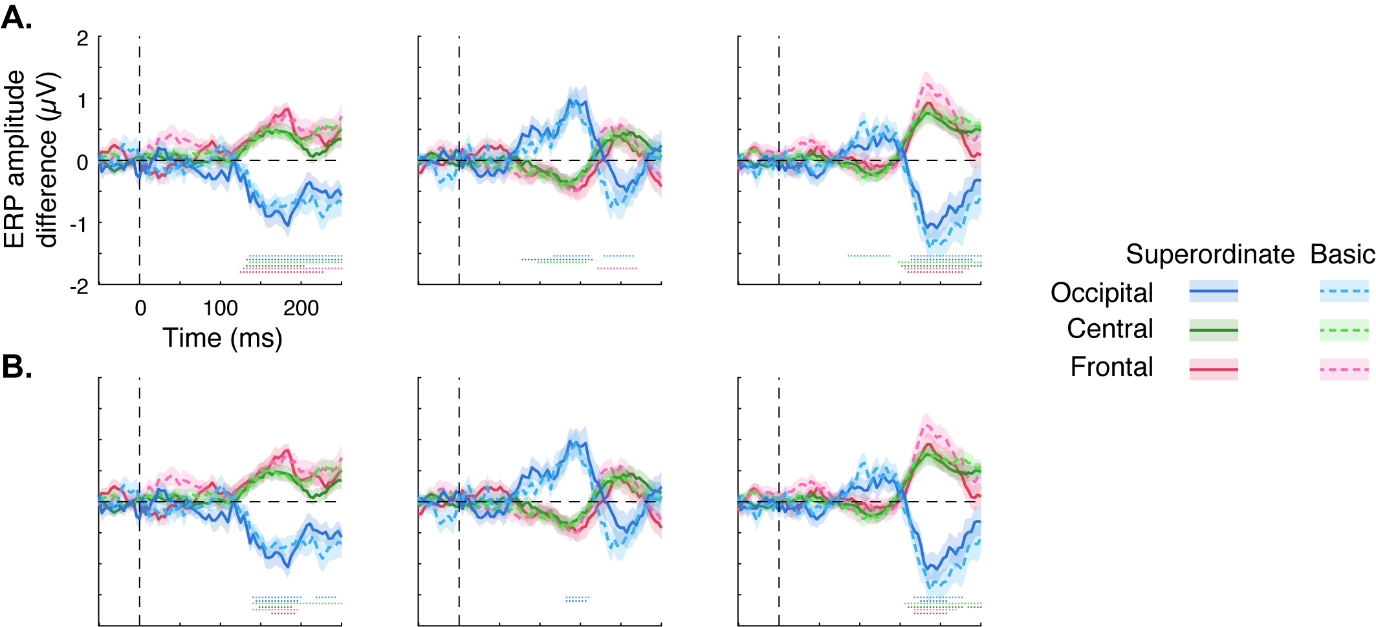


Supplementary Figure 4. Results of the ERP analyses using unfiltered data. Each line plots the difference in responses between two categories in a given categorisation task averaged over electrodes located at occipital, central, and frontal sites. Shaded areas around the mean amplitude represent SEM. Significant differences are represented with matching-coloured dots at the bottom of the figures using cluster-based permutation tests (A) or paired t-tests with p<.005 for at least 15 ms (B).

### Variability of results with different sample sizes

To test the robustness of our results with our sample size, we iteratively sampled 4 to 17 participants with replacement from our original sample of 17. For each sample we conducted paired sample t-tests on decoding performance (results vs shuffled baseline) at each timepoint and each category pair and categorisation task. We noted any significant differences (p<.05). We computed this 1000 times and assessed the proportion of these iterations that yielded a significant difference for that timepoint, category pair, and indicating the power given the sample size. The results (Fig. S5) are stable and indicate high power from 10 participants onwards. This suggests that our sample size of 17 was more than adequate to address our question.

Supplementary Figure 5. Proportion of significant paired t-tests for decoding pairs of categories at the superordinate (top-row) or basic (middle row) level. The last row represents the effect of task (difference between superordinate and basic level decoding, top and middle rows).

## ERP results

The ‘raw’ (not differential) EEG responses to each of the three categories of interest (bird, non-bird animal, and vehicle) in the basic and superordinate categorisation tasks are represented in Fig. S6. The results show similar, high ERP amplitudes in all conditions Early (from 100 ms) bilateral response in occipital electrodes increase over time (200-250 ms), accompanied by negative polarity in frontal electrodes. Amplitudes then decrease and become relatively weak and more centrally located by 450 ms.


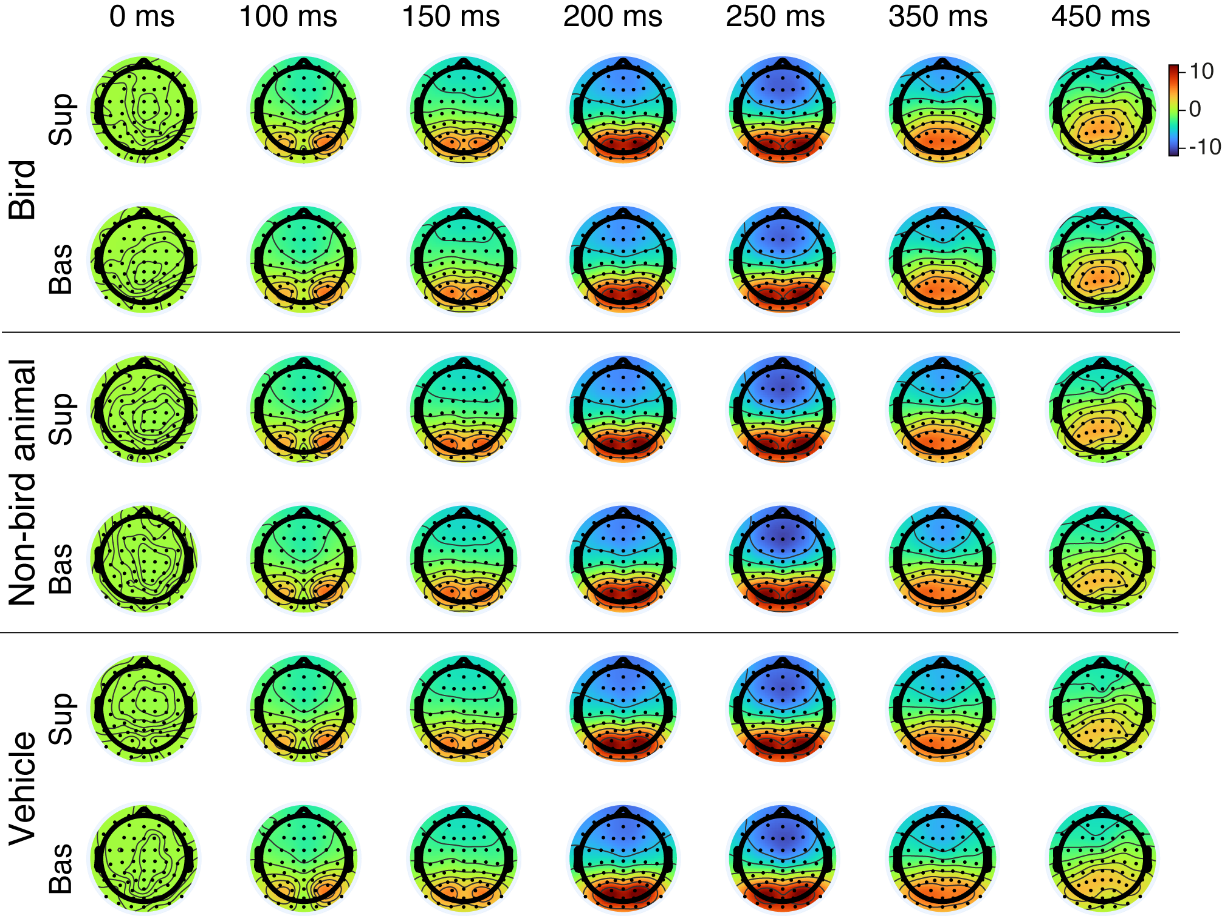


Supplementary Figure 6. ERP amplitude (in µV) for each of the three categories of interest (bird, non-bird animal and vehicle) in the basic and superordinate categorisation tasks.

In the main text we report differential ERP waveforms between pairs of categories pooled over specific electrodes at occipital, central and frontal locations, between -50 ms to 250 ms. These figures follow the same parameters as in previous reports (VanRullen and Thorpe 2001) to facilitate comparison. In Fig. S7 we report the same differential ERP responses for each individual electrode over the full time window. The topographies of the same results can be found in section 2.4 of the Supplementary results.


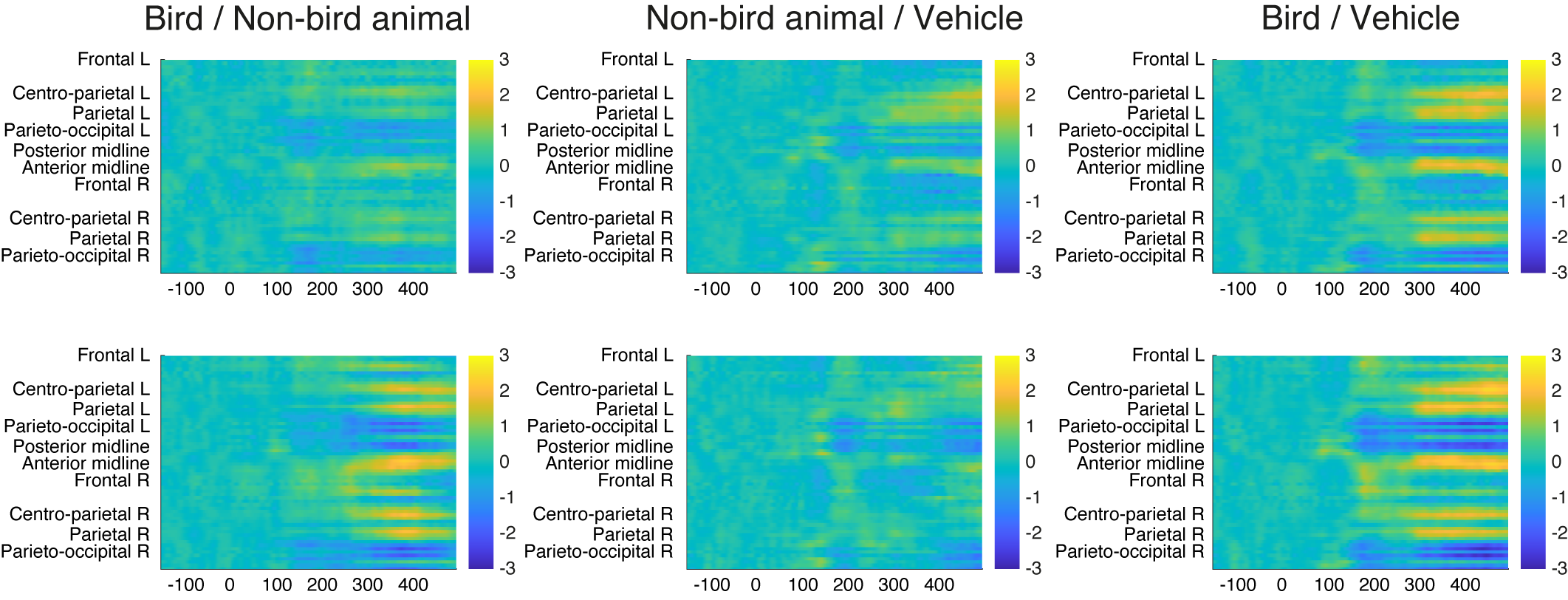


Supplementary Figure 7. Differential ERP amplitude (in µV) between pairs of categories in the superordinate (top row) and the basic (bottom row) categorisation task. Each line represents the differential ERP amplitude of one electrode located in the left (L) or right (R) hemisphere.

## MVPA analyses based on correct trials

We reran the decoding analysis using only correct trials. Decoding performance is highly similar as when all trials were included (Fig. S8). However, significant differences arise at slightly later timepoints especially while discriminating bird and non-bird animal categories. The precise significant decoding onsets depend on the statistical analyses used to control for multiple comparisons. Here the significant onsets for correct-trials are later than for all-trials when cluster-based permutation tests are used, but earlier than when p<.005 for at least 15 consecutive ms was used as a criterion.


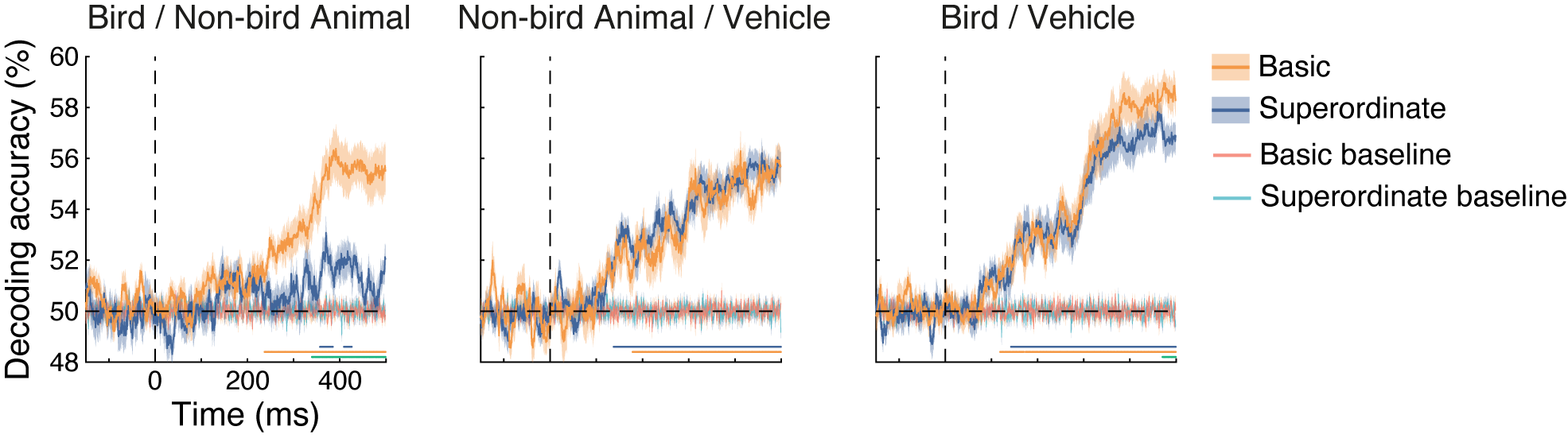


Supplementary Figure 8. Decoding analysis as in Fig. 3 based only on correct trials.

## Topographies and source localisation using differential ERPs

To determine the electrodes involved in discriminating pairs of categories, we used a functional source localisation method based on EEG templates (Poncet and Ales 2023). In the main text, we applied this method to the LDA classifier’s weights. Because we used an LDA classifier, the topographies of the classifier’s weights and therefore the source localisation results, should be equivalent to the differential ERP response for each of the six pairs of conditions (Haufe et al. 2014). To confirm this, we present below the topographies (Fig. S9A) of the differential ERPs between pairs of categories and apply the functional source localisation method to these differential ERPs. As expected, the topographies are similar to the topographies of the classifier’s weights and the retrieved functional sources (Fig. S9B) are also mostly identical to the ones reported in the manuscript with some very minor differences possibly due to sampling differences when running the decoding analysis.


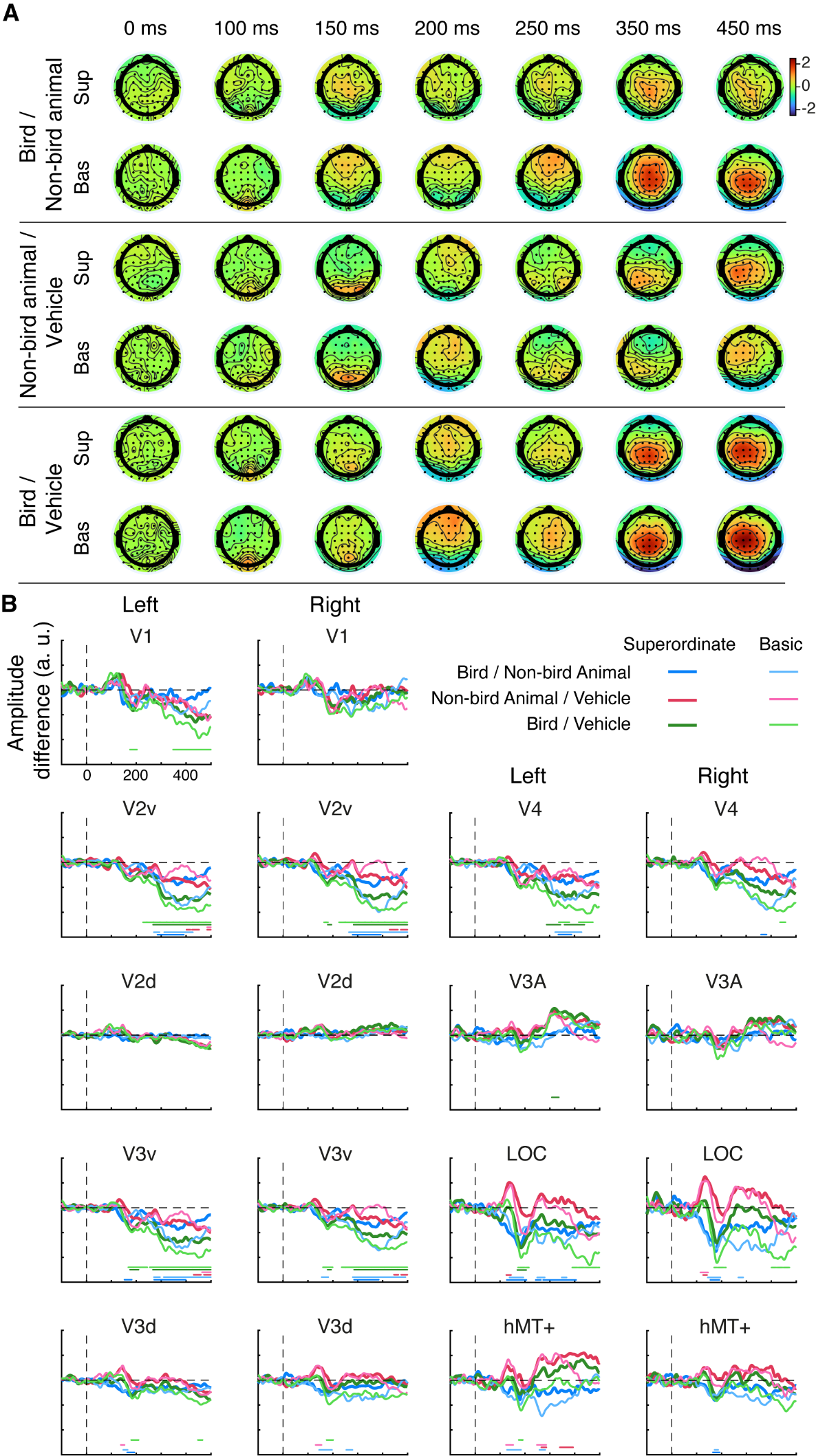


Supplementary Figure 9. (A) Topographies of the differential ERPs (in µV) between pairs of categories in the basic (Bas) and superordinate (Sup) categorisation tasks. (B) Time course of the brain activity in visual areas for discriminating pairs of object categories. Significant differences with a corresponding null distribution are represented with matching coloured dots at the bottom of the figures.

# References

Benjamin DJ, Berger JO, Johannesson M, Nosek BA, Wagenmakers E-J, Berk R, Bollen KA, Brembs B, Brown L, Camerer C, et al. 2018. Redefine statistical significance. Nat Hum Behav. 2(1):6–10. https://doi.org/10.1038/s41562-017-0189-z

Haufe S, Meinecke F, Görgen K, Dähne S, Haynes J-D, Blankertz B, Bießmann F. 2014. On the interpretation of weight vectors of linear models in multivariate neuroimaging. Neuroimage. 87:96–110. https://doi.org/10.1016/j.neuroimage.2013.10.067

Poncet M, Ales JM. 2023. Estimating neural activity from visual areas using functionally defined EEG templates. Human Brain Mapping.:hbm.26188. https://doi.org/10.1002/hbm.26188

Rousselet GA. 2025. Using cluster-based permutation tests to estimate MEG/EEG onsets: How bad is it? Eur J Neurosci. 61(1):e16618. https://doi.org/10.1111/ejn.16618

Sassenhagen J, Draschkow D. 2019. Cluster-based permutation tests of MEG/EEG data do not establish significance of effect latency or location. Psychophysiology. 56(6):e13335. https://doi.org/10.1111/psyp.13335

Tanner D, Morgan-Short K, Luck SJ. 2015. How inappropriate high-pass filters can produce artifactual effects and incorrect conclusions in ERP studies of language and cognition. Psychophysiology. 52(8):997–1009. https://doi.org/10.1111/psyp.12437

VanRullen R. 2011. Four Common Conceptual Fallacies in Mapping the Time Course of Recognition. Frontiers in Psychology [Internet]. [accessed 2013 Jul 26] 2. https://doi.org/10.3389/fpsyg.2011.00365

VanRullen R, Thorpe SJ. 2001. The time course of visual processing: from early perception to decision-making. J Cogn Neurosci. 13(4):454–461.
